# Accelerated start-up, long-term performance and microbial community shifts within a novel upflow porous-plated anaerobic reactor treating nitrogen-rich wastewater *via* ANAMMOX process

**DOI:** 10.1039/c9ra04225c

**Published:** 2019-08-21

**Authors:** Dachao Zhang, Shi Xu, Philip Antwi, Longwen Xiao, Wuhui Luo, Zuwen Liu, Jianzheng Li, Hao Su, Cheng Lai, Frederick Ayivi

**Affiliations:** Jiangxi University of Science and Technology, School of Resources & Environmental Engineering, Jiangxi Key Laboratory of Mining & Metallurgy Environmental Pollution Control Ganzhou City Jiangxi province 341000 PR China kobbyjean@yahoo.co.uk; Harbin Institute of Technology, State Key Laboratory of Urban Water Resource and Environment, School of Environmental 73 Huanghe Road Harbin 150090 P. R. China; Fayetteville State University, Department of Geography 1200 Murchison Road Fayetteville NC 28301 USA

## Abstract

The anaerobic ammonium oxidation (anammox) process has gained much popularity in recent years following its success in nitrogen removal. However, not much has been reported on techniques to promote anammox bacteria immobilization and associated microbial community evolution. In this study, a novel upflow porous-plate anaerobic reactor (UPPAR) was developed and explored to promote biomass (anammox) retention and growth. To comprehend the performance of the UPPAR, its nitrogen removal efficiencies, as well as the microbial community dynamics involved in the nitrogen removal process, was evaluated and reported. When NLR ranging 0.98–1.08 kg m^−3^ d^−1^ was introduced at various stages of the UPPAR operation, a rapid start-up was achieved in 63 d, and the overall nitrogen removal rate could reach 90–95%. By the end of the start-up period, it was revealed that Proteobacteria abundance had reduced by 43.92% as opposed Planctomycetes which increased from 2.95% to 43.52%. Conversely, after the UPPAR had been operated for 124 d, thus at steady-state, the most pronounced phylum observed was Planctomycetes (43.52%) followed by Proteobacteria (26.63%), Chloroflexi (5.87%), Ignavibacteriae (5.55%), and Bacteroidetes (4.9%). Predominant genera observed included *Candidatus Kuenenia* – (25.46%) and *Candidatus Brocadia* – (3.15%), an indication that nitrogen removal mechanism within the UPPAR was mainly conducted *via* autotrophic anammox process. Scanning electron microscopy (SEM) revealed that sludge samples obtained at steady-state were predominantly in granular form with sizes ranging between 2 mm to 5 mm. Granules surfaces were dominated with normal to coccoid-shaped cells as revealed by the SEM.

## Abstract 2

Schematic diagram of the upflow porous-plate anaerobic reactor and nitrogen removal pathways occurring within the reactor.

## Graphical abstract

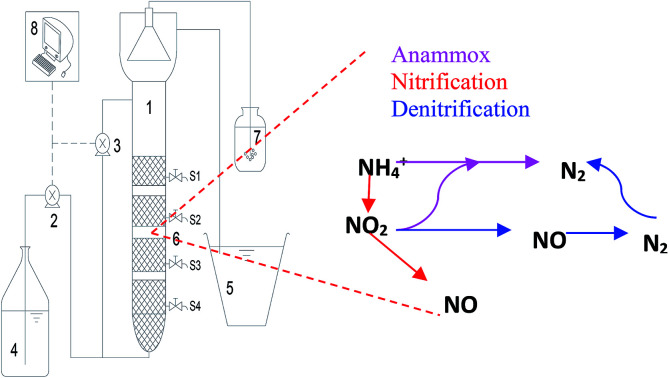

## 1. Introduction

The concept of industrialization has gained extensive attention in recent years following its significant contribution in a country's development and improvement of economies or the lives of people. However, industrialization and its related activities have always affected many key natural resources including environment, water bodies, land, and air. For instance, industrial activities generate huge volumes of wastewater containing significant concentrations of nitrogen, carbon, and phosphorus. Wastewater characterized by these pollutants and nutrients often leads to water eutrophication and impact negatively on both human and aquatic life. Therefore, wastewater (industrial, domestic or municipal) treatment is no doubt, an important agenda that requires extensive promotion to avoid environmental pollution and its related consequences. Although government-mandated institutions are enforcing pollution laws, a more stringent discharge standard is also paramount to achieving a healthy water life across the globe.^1^

Nitrogen removal *via* traditional biological processes (nitrification and denitrification) has always engaged the activities of aerobic nitrification and anaerobic denitrification supported by both autotrophs and heterotrophs.^2^ However, the carbon source for denitrification is one key component necessary to facilitate the reduction of nitrate and nitrite to gaseous nitrogen. Supplementation of organic carbon source (external/*in situ*) or enhancing C/N ratio by decreasing NH_4_^+^-N concentration through a chemical process has been exploited successfully over the years during nitrogen removal from wastewater, but this practice has been recorded as a relatively expensive approach for a commercial scale treatment plant. Autotrophic nitrogen removal by anaerobic ammonium oxidation (anammox) process has emerged due to its cost-efficiency, high-performance efficiency in the absence of organic carbon sources, environmentally friendly, and low energy consumption. Anammox process has highly been recommended by a significant number of researchers mainly due to the successes the process has achieved so far.^3,4^ Apart from the numerous advantages associated with the anammox process, the concept of low or minimal sludge yield has been one key issue that has often fueled the global acceptance of the anammox process as opposed to the traditional process by heterotrophic bacteria.^5^

Conversely, anammox process, specifically its responsible bacteria is characterized as slow growing bacteria.^5^ This consequently leads to slow or long periods of startups and poor nitrogen removal efficiency. Also, anammox biomass is highly sensitive to several environmental conditions including upflow velocity, temperature, pH, dissolved oxygen (DO), and other chemical inhibition. Therefore, factors necessary to enhance anammox biomass growth is necessary to be established and implemented in order to achieve outstanding results.^6,7^ Anammox process has successfully been explored in many traditional bioreactors including sequencing batch reactor (SBR),^8,9^ membrane bioreactor (MBR)^10^ and upflow anaerobic sludge blanket (UASB).^11^ Although SBR has an excellent biological retention capacity, it requires a high degree of automation to control filling, stirring, reaction, decanting and aeration.^12^ To best of our comprehension, not much has been reported on strategies to enhance anammox process start-up, selection of appropriate sludge for inoculation as well as bioreactor design that will seek to enhance anammox biomass retention.

Considering anammox bacteria and its slow growth rate, sludge retention or biomass immobilization will contribute significantly to reducing anammox process start-up periods and long-term performance. For instance, sludge retention capabilities of an UASB is weak due to the production of N_2_ gas which often floats and washes out weightless sludge (particularly anammox bacteria) or sludge that seems to take a longer time to settle out of suspension.^13^ In addition, MBR has achieved many success stories as well but the installation and running of MBR have widely been reported as an expensive technology due to the frequent replacement of blocked membranes in order to sustain performance efficiency.^14^ Therefore, to establish an efficient, accelerated startup and robust anammox process, prudent measures are necessary to help prevent sludge lost. So far, gel immobilization methodology has been widely employed to immobilize autotrophic nitrifying bacteria and anammox bacterial^15–17^ and this has demonstrated some level of good efficiency but its long-term sustainability and applicability at industrial/commercial scale have always been a challenge. In this regard, the development of new ways to simultaneously enhance the anammox bacteria growth rate as well as enhancing their retention in a reactor will be significantly beneficial to the industrial application of the anammox technology. To best of our knowledge, the novel porous-plate anammox biomass immobilization technique proposed in this study has not yet been exploited and reported. Besides strategies for sludge retention, a detailed investigation of microbial community evolution is also paramount in establishing the success of the proposed novel biomass retention strategy. Although many conventional molecular tools have been developed for microbial communities studies,^18–20^ high throughput gene sequencing methodology has been highly recommended based on its high efficiency and its potential for more in-depth and more detailed investigations into complex microbial communities.^20,21^

In this study, a novel upflow porous-plate anaerobic reactor (UPPAR) was proposed, developed and explored for anammox biomass immobilization and accelerated start-up during nitrogen-rich rare-earth mining wastewater treatment *via* anammox process. To establish the efficacy of the proposed strategy conducted within the UPPAR, nitrogen removal efficiencies at start-up and long-term was studied and reported accordingly. Furthermore, the microbial community evolution (by high throughput gene sequencing – Illumina) and sludge morphology in the inoculum and acclimatized sludge in the UPPAR were investigated to provide insights into the biological mechanism within the UPPAR. This study is expected to provide useful knowledge on (1) the effects of the novel UPPAR on microbial community evolution, (2) strategies to enhance startup and steady-state performance.

## 2. Materials and methods

### 2.1 Characteristics of simulated rare-earth wastewater

Rare-earth mining wastewater was simulated in accordance with that reported in literature^52,53^ in the following composition (g L^−1^): KH_2_PO_4_ 0.010, MgSO_4_·7H_2_O 0.030, CaCl_2_ 0.004, KHCO_3_ 1.250 and 1.25 mL L^−1^ of trace elements (I and II) solution. NH_4_^+^-N and NO_2_^−^-N were supplied by NH_4_Cl and NaNO_2_, respectively, as needed. The influent pH was adjusted using HCl and NaOH. Trace element I contained the following in (g L^−1^): EDTA 5, FeSO_4_, whereas that in trace element II (g L^−1^) were: EDTA 15, ZnSO_4_·7H_2_O 0.43, CuSO_4_·5H_2_O 0.25, NiCl_2_·6H_2_O 0.19, MnCl_2_·4H_2_O 0.99, CoCl_2_·6H_2_O 0.24, NaMoO_4_·2H_2_O 0.22, H_3_BO_4_ 0.014.

### 2.2 Experimental setup and operation

**Fig. 1:**
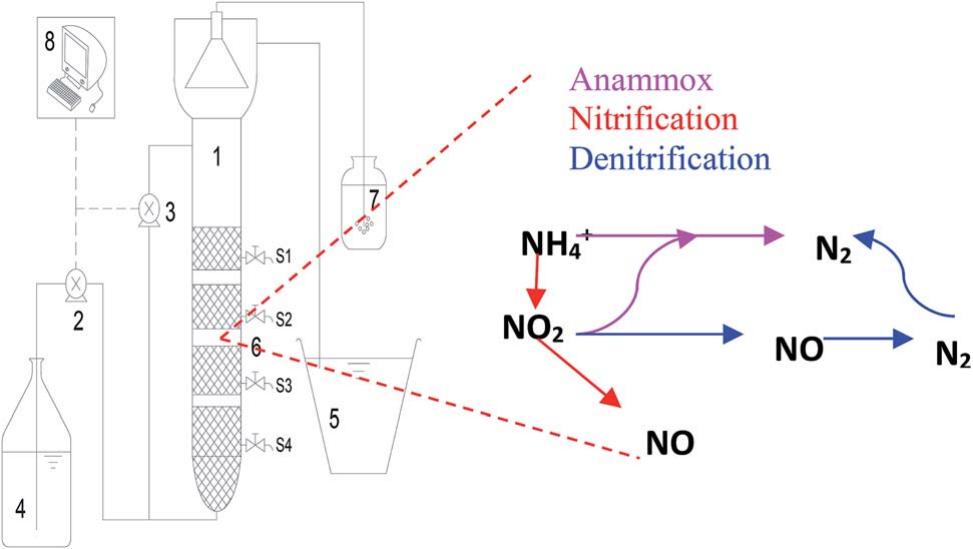
Schematic diagram of the upflow porous-plate anaerobic reactor and nitrogen removal pathways occurring within the reactor: (1) settling zone; (2) influent pump; (3) recycling pump; (4) influent tank; (5) effluent tank; (6) sampling port; (7) water seal; (8) online DO, ORP and temperature control system.

The upflow porous-plated anaerobic reactor (UPPAR) was constructed with transparent Plexiglas (Fig. 1). The UPPAR had an internal diameter, effective height and an effective working volume of 40 mm, 950 mm and 1.2 L, respectively. The entire reaction zone was divided into four zones along with the vertical height by integrating porous plates at specified depths aimed at enhancing sludge retention in the reactor (Fig. 1). The temperature of the reactor was maintained at mesophilic condition (35 °C) using a hot water bath and thermistor. The reactor was clad in black cloth to avoid the evolution of phototrophic bacteria and photo-inhibition on the anammox consortium. A peristaltic pump (Langer Instruments, BT10032J, UK) was used to feed the simulated wastewater to the reactor. After the required operational hardware had been successfully installed for the operation of the UPPAR, anammox sludge and aerobic sludge were collected from a nearby wastewater treatment plant to inoculate the UPPAR. The anammox sludge and aerobic sludge were mixed in a ratio of 1 : 5 (mixed liquor suspended solids (MLSS), and mixed liquor volatile suspended solids (MLVSS) was 4.32 and 3.52 g L^−1^ respectively) and used as the inoculum in the UPPAR. The experiment conducted in this study comprised of four stages (I, II, III, and IV) and the operating parameters associated with each stage is presented in Table l. After sludge had been acclimatized with environmental conditions, high nitrogen loading rate was fed to the reactor whilst maintaining NH_4_^+^-N : NO_2_^−^-N ratio at 1 : 1.32. NH_4_^+^-N and NO_2_^−^-N was supplied to the simulated wastewater using NH_4_Cl and NaNO_2_, respectively.

**Table 1:** UPPAR operating conditions and stoichiometric ratios (ΔNH_4_^+^ : ΔNO_2_^−^ : ΔNO_3_^−^)

Phase	Stage	Time (d)	HRT (h)	Av. NH_4_^+^-N (mg L^−1^)	Av. NO_2_^−^-N (mg L^−1^)	Average NLR (kg m^−3^ d^−1^)	ΔNH_4_^+^ : ΔNO_2_^−^ : ΔNO_3_^−^ ratio
Start-up	I	1–6	10	51.95	62.5	0.27	1 : 0.37 : (−1.09)
	II	7–23	10	50.42	62.5	0.27	1 : 0.94 : 0.16
	III	24–63	10	184.27	225.87	0.98	1 : 1.25 : 0.24
Long term	IV	64–124	10	200.84	250.34	1.08	1 : 1.29 : 0.25

### 2.3 Analytical methods

All physico-chemical parameters in the water samples were determined daily in line with the protocols established in standard methods.^22^

Ammonium (NH_4_^+^-N), nitrite (NO_2_-N) and nitrate (NO_3_-N) were determined with a spectrophotometer (UV-1800 UV-VIS, Shimadzu Corporation, Japan) at a wavelength of 420 nm, 540 nm, and 220 nm, respectively. Influent, effluent and reactor pH was determined online using a precision ion meter (Zhengzhou Nanbei Instrument Equipment Co., Ltd., PXS-450). Granular sludge particle size was measured after photographing with a Motic biological microscope and Image-Pro Plus.^23^ Nitrogen loading rate (NLR), nitrogen removal rate (NRR), and all stoichiometric ratios including ammonium removed (ΔNH_4_^+^), nitrite consumed (ΔNO_2_^−^) and nitrate produce (ΔNO_3_^−^) were estimated with eqn (1), (2), (3), (4) and (5), respectively.

1 NLR = [NH_4_^+^-N]_Inf_/HRT

2 NRR = ([NH_4_^+^-N]_Inf_/HRT) − ([NH_4_^+^-N]_Eff_/HRT)

3 ΔNH_4_^+^ = ([NH_4_^+^-N]_Inf_) − ([NH_4_^+^-N]_Eff_)

4 ΔNO_2_^−^ = [NO_2_-N]_Inf_ − [NO_2_-N]_Eff_

5 ΔNO_3_^−^ = [NO_3_-N]_Eff_ − [NO_3_-N]_Inf_

where [NH_4_^+^-N]_Inf_, influent ammonium; HRT, hydraulic retention time; [NH_4_^+^-N]_Eff_, effluent ammonium; [NO_2_-N]_Inf_, influent nitrite; [NO_2_-N]_Eff_, effluent nitrite; [NO_3_-N]_Eff_, effluent nitrate; and [NO_3_-N]_Inf_, influent nitrate.

### 2.4 DNA extraction, PCR amplification, and sequencing cluster analysis

The inoculum (X_0_) and the sludge obtained at the end of some of the proposed stages including end of stage II (X_II_), stage III (X_III_), and stage IV (X_IV_) were investigated to establish microbial community succession. Bacteria DNA Isolation Kit manufactured by MOBIO Laboratories, Inc., USA, was used to extract total DNA of the samples.^52^ After agarose gel electrophoresis,^24^ genomic DNA was quantified for PCR reaction where universal primers were exploited to amplify genomic DNA samples.^20^ The proposed PCR amplification was conducted in Eppendorf Mastercycler under the condition described by Yuan and coworkers.^25^ Furthermore, 1% agarose was also employed to amplify products, analyzed and finally sequenced qualified DNA using the Illumina Miseq sequencing platform. Primer sequences, short sequences, and low-quality sequences were removed after the sample reads had been filtered. Detailed cluster and statistical analysis were conducted as described by Antwi and coworkers.^20^ The BLAST tool of the National Center for Biotechnology Information (NCBI) was employed to search and compare^24^ gene sequences. Finally, nucleotide sequences of dominant abundance were deposited in the NCBI GenBank.

### 2.5 Sludge morphology and characterization

The sludge samples obtained from the reactor and that of the inoculum were also subjected to further investigations to unravel at differences in morphology. The collected sludge samples were initially pretreated before a morphological study was initiated. The pretreatment was conducted by removing impurities within sludge samples. Thus, samples were washed in deionized water after they had been separated in 10 mL aliquots.^26^ The samples were then fixed overnight with 2.5% vol/vol glutaraldehyde (pH 6.8) at 4 °C after which each sample was rinsed twice with ultra-pure water and again dehydrated with ethanol solutions at 25, 50, 70, 80, 90, and 100%.^6^ The morphology of the pretreated sludge samples was then viewed with environmental scanning electronic microscope (FEI Quanta-200).

## 3. Results and discussion

### 3.1 Start-up performance of the UPPAR

**Fig. 2:**
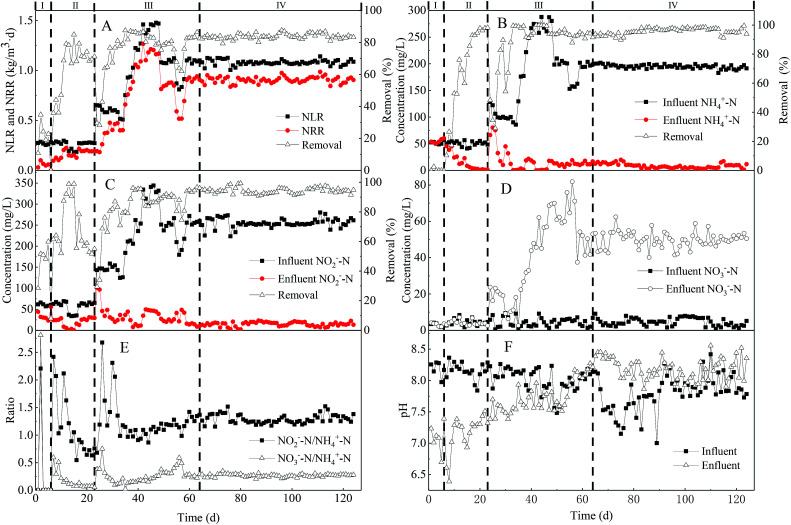
Start-up and long-term performance of the UPPAR: (A) nitrogen loading and removal rate; (B) NH_4_^+^-N removal efficiency; (C) NO_2_^−^-N uptake efficiency; (D) nitrate production and accumulation; (E) stoichiometric ratio; (F) pH profile.

A rapid start-up was established within the UPPAR treating rare-earth mining wastewater after 63 d of operation. As illustrated in Fig. 2, the start-up period of the UPPAR comprised of three main stages *viz.*, bacteria cell lysis (stage-I), sludge acclimatization (stage-II) and enhanced sludge activity (stage-III). As NLR of 0.27 kg m^−3^ d^−1^ (influent NH_4_^+^-N and NO_2_^−^-N concentration of 51.95 mg L^−1^ and 62.50 mg L^−1^, respectively) was employed in the UPPAR in stage-I, it was found that effluent NH_4_^+^-N concentration was relatively higher compared to that in the influent (Fig. 2B). This phenomenon mainly suggested bacteria cells had undergone some form of lysis whilst adapting to the new environment introduce in the UPPAR. Thus, some microorganisms in the inoculum were unable to adapt to the UPPAR operational conditions which subsequently led to cell death and release of ammonia in the effluent. Notably, this observable phenomenon manifested only within the first six days after the UPPAR was started-up. A similar observation was reported by Wang and coworkers whiles conducting a study on the startup of the anammox process within an UASB reactor.^27^ In their study, it was established that the concentration of NH_4_^+^-N in the initial effluent was significantly greater compared to the influent.

However, NO_2_^−^-N concentration in the effluent was 30 mg L^−1^, an indication that 51.8% of NO_2_^−^-N removal was achieved during stage-I (Fig. 2C). The performance in term of nitrogen removal observed in stage-I was mainly attributed to the presence of organic matter (COD from inert biomass), nitrifying bacteria (convert NO_2_^−^-N to NO_3_^−^-N) and the final denitrification reaction by denitrifying bacteria which converted NO_3_^−^-N to N_2_ in the presence of the organic carbon *via*. Notably, NO_2_^−^-N removal increased with time as shown in Fig. 2C in stage-II (7^th^–23^rd^ day), although influent NH_4_^+^-N, NO_2_^−^-N concentrations as well as HRT and NLR were maintained (Table 1), simultaneous removal of NH_4_^+^-N (Fig. 2B) and NO_2_^−^-N (Fig. 2C) was noticed on the 7^th^ day. However, the removal efficiency of NH_4_^+^-N was relatively poor as NH_4_^+^-N removal rate reached was only 12% whereas NO_2_^−^-N could reach 61.5%. In both reported stages (I and II), NO_3_^−^-N was not observable in the effluent (Fig. 2D) indicating denitrification and anammox reactions were in coexistence within the UPPAR at stages-I and II. It was also believed that those inert and unwanted bacteria gradually washed out of the UPPAR and thus, electron acceptor bacteria population might have reduced giving rise to the evolution and growth of the anammox bacteria. By the 17^th^ day, NH_4_^+^-N removal was remarkably enhanced to about 94.77% and only 3.89 mg L^−1^ of NH_4_^+^-N was determined in the effluent (Fig. 2B). Similarly, NO_2_^−^-N removal rate was also elevated along with the NH_4_^+^-N observed on day 17. From the 18^th^ to 23^rd^, NH_4_^+^-N removal rate continued to be above 95%. Although effluent NH_4_^+^-N and NO_2_^−^-N concentration decreased significantly during stage II, it was however found that pH of the effluent (Fig. 2F) was higher compared to that in the influent, an indication that anammox bacteria population was increasing within the UPPAR.

The performance of the novel UPPAR in terms of nitrogen removal was more noticeable during stage III (24^th^ day to 63^rd^ day). In stage-III, HRT was maintained whereas NLR was varied anytime a steady-state was achieved with the previous NLR. Herein, HRT of 10 h was maintained and NLR elevated from 0.27 kg m^−3^ d^−1^ (stage-I and II) to 0.98 kg m^−3^ d^−1^ (stage-III), thus by increasing influent NH_4_^+^-N and NO_2_^−^-N concentration to 184.27 mg L^−1^ and 225.87 mg L^−1^, respectively. As NLRS of 0.98 kg m^−3^ d^−1^ was employed in stage-III, the performance of the UPPAR was significantly improved (Fig. 2B). NH_4_^+^-N removal rate reached on the 33^rd^ day was about 99%. After day 38, NO_2_^−^-N removal could reach 90.16% (Fig. 2C), confirming efficient removal of NH_4_^+^-N and NO_2_^−^-N by the UPPAR. Conversely, NO_3_^−^-N accumulation commenced showing up in stage-III (Fig. 2D). However, per the mass balance and stoichiometry of the anammox reaction given in eqn (6), a majority of the observable accumulated NO_3_^−^-N within the UPPAR was obtained from the anammox reaction and whereas the remaining fraction is believed to have come from the nitrite-oxidizing bacteria (NOB) that coexisted with the anammox bacteria. Furthermore, the ratio of NH_4_^+^-N removal and NO_2_^−^-N removal observed in stage (III) increased consistently (Fig. 2E), an indication that anammox bacterium had become the dominant population within the UPPAR that anammox had become the dominant as shown in Fig. 2E, whiles NRR of 0.94 kg m^−3^ d^−1^ was achieved by the UPPAR, the ratio of NH_4_^+^-N, NO_2_^−^-N removal and NO_3_^−^-N formation as at day 63 was 1 : 1.32 : 0.22 which agreed well with the theoretical value (1 : 1.32 : 0.26). The estimated ratio further confirmed that anammox bacteria were predominant among the microbial communities within the UPPAR. Besides the efficient performance of the UPPAR, it was apparent that no sludge washout occurred, suggesting that the UPPAR and its features including the porous-plate integrated within the UPPAR, had promoted an accelerated growth, prevented sludge washout and finally quickened the start process in this study. Consequently, a rapid start-up was achieved within 63 days of the UPPAR operation. The fast start-up associated with the UPPAR was attributable to the porous-plates incorporated in the UPPAR.

Literature reveals that efficient and fast start-up of the anammox process using traditional bioreactors has always remained the major bottleneck and a subject of great concern. Chamchoi and coworkers operated an anammox process using SBR inoculated with conventional aerobic activated sludge.^6^ Anammox activity was established after the SBR had operated for over 120 days. In another study, Lü and coworkers employed MBR and anaerobic baffled reactor (ABR) reactors inoculated with nitrifying sludge and investigated how fast a rapid startup of the anammox process could be obtained.^28^ In their report, it was established that MBR and ABR achieved a successful startup within 90 d and 111 d, respectively. Compared to these published reports, it was evident that, the UPPAR had experienced a rapid start-up and that the use of the porous plate anaerobic reactor has certain advantages over the conventional bioreactors. As demonstrated in the anammox reaction given in eqn (6),^29^ it could be deduced that typical anammox reaction consumes acidity, leading to an increase in pH in the reactor.

6 NH_4_^+^ + 1.32NO_2_^−^ + 0.066HCO_3_^−^ + 0.13H^+^ →0.26NO_3_^−^ + N_2_ + 0.066CH_2_O_0.5_N_0.5_ + 2.03H_2_O

Therefore, a specific range of pH is deemed necessary for efficient start-up and long-term operation of an anammox process. Ideally, pH inhibits anammox bacteria performance, particularly during the start-up process. Studies have demonstrated that the optimum pH range for the anammox process is 7.5–8.3, where 8.3 is the optimum working pH. In this study, pH in the influent was mainly adjusted by NaHCO_3_ addition leading to a fluctuated pH which ranged 7.7 and 8.3 during the start-up process. As illustrated in Fig. 2F, fluctuation of both influent and effluent pH was more pronounced during the commencement of the UPPAR operation, thus during the cell lysis phase (stage I). However, effluent pH (pH = 7) was significantly low as opposed to pH (pH = 8.3) of the influent. In stage (II), the effluent pH started increasing, although relatively lower as opposed to that of the influent (Fig. 2F). The variation in influent and effluent pH revealed the existence of anammox bacteria except that their relative abundance was not that significant. Inversely, as anammox activities started to improve in stage III, the variation in influent and effluent pH reduced significantly (Fig. 2F). At this time, the average effluent pH reached 7.7 whereas that in the influent was 8.3. Whilst the operation of stage-III progressed, the anammox process improved significantly in the later periods of stage (III), indicating dominance or growth among anammox bacteria population.

### 3.2 Accelerated start-up of anammox activity by porous-plate

Gel has extensively been employed to immobilize anammox biomass in the form of beads. However, to the best of our knowledge, a physical approach employed to achieve the same anammox biomass immobilization has not been exploited. In this study, a porous-plate was integrated within the UPPAR at various depths to enhance anammox biomass immobilization. As presented in Table 2, several reports are put out there when gel material was employed to immobilize anammox biomass in order to enhance and quicken anammox activities (Table 2). Compared to other biomass immobilization strategies including immobilized gel, bamboo char and hollow fiber (Table 2), it was observed that an accelerated start-up was achieved with the UPPAR within 63 days of operation (in this study). The study further suggested that high retention of anammox biomass was feasible by the porous-plate. Also, although mixed consortium was used as inoculum in the UPPAR, the efficiency of the porous plate was affirmed by the dominance of genus *Candidatus Kuenenia* that was more pronounced within the UPPAR. Compared to naturally aggregated biomass into granules, a report found in the literature revealed that high TN removal is mostly achieved when gel or manmade material are employed as biomass immobilization agents. For instance, when gel beads were employed to immobilize biomass with a concentration of 3.8 g_VSS_ L^−1^, NRR of 8.2 kg N m^−3^ d^−1^ was achieved in just 100 days, whereas the UPPAR containing biomass with a concentration of 3.52 g_VSS_ L^−1^ could only achieve NRR of 0.92 kg N m^−3^ d^−1^. The remarkable performance of the anammox gel beads was possible as a result of (1) higher relative effective diffusivity as opposed to granulated biomass^30^ and (2) inability of inner biomass of granules to remain metabolically active. Although, the gel immobilization methodology has proved to be efficient in TN removal, its tendency to achieve a rapid startup so far as the anammox process is concerned and this calls for a deeper investigation to elucidate its ability to withstand hydrodynamic behavior.

**Table 2:** Comparison of immobilized methods for anammox bacteria retention^a^

Start-up (day)	Dominant genera	Biomass (g_VSS_ L^−1^)	NRRs (kg N m^−3^ d^−1^)	Reactor type	Temp (°C)	Biomass retention	Reference
100	*Ca. J. caeni*	3.80	8.20	CSTR	33	Immobilize gel	45
100	Not reported	0.10	4.40	CSTR	30	Immobilize gel	46
67	*Ca. J. caeni*	0.55	3.70	CSTR	36	Immobilize gel	47
180	Mixed sludge	0.32	1.69	CSTR	34	Immobilize gel	48
65	*Ca. J. caeni*	1.34	3.80	CSTR	30	Immobilize gel	49
85	NR	11.33	NR	UASB	30	Bamboo char	50
75	*Ca. Brocadia*	4.90	NR	CAMBR	13	Hollow fiber	51
90	*Ca. Jettenia*	4.90	NR	CAMBR	13	Hollow fiber	51
63	*Ca. Kuenenia*	3.52	0.92	UPPAR	35	Porous plate	This study

a CSTR, continuous stirred tank reactor; UASB, upflow anaerobic sludge blanket; CAMBR, combined ABR and MBR; NR, not reported.

### 3.3 Steady-state performance of the UPPAR

In stage (IV), influent NLR was elevated to about 1.08 kg m^−3^ d^−1^, and the efficiency of the reactor was further investigated. Stage (IV) lasted for 60 days, with an influent NH_4_^+^-N and NO_2_^−^-N concentrations of 200 mg L^−1^ and 250 mg L^−1^, respectively, fed to the UPPAR. At steady-state in stage-IV, NH_4_^+^-N and NO_2_^−^-N removal rates reached 95% and 94%, respectively, indicating the stability of the UPPAR performance (Fig. 2B). In reference to the NRR, it was estimated that NRR of 0.92 kg m^−3^ d^−1^ was achieved which suggested that removal rates attained stability of 84.3%. NO_3_^−^-N concentration in the effluent could reach 50 mg L^−1^, indicating NO_3_^−^-N in the effluent increases based on substrate supply or dosed and anammox bacteria enrichment. Therefore, a more robust and NOB out-selection or inhibition strategy would be very necessary to enhance the TN removal within the UPPAR. At the steady-state (stage IV), effluent pH was relatively higher than the influent (Fig. 2F). Zhang and coworkers reported that traditional anammox reaction consumes acidity, and thus, this often leads to an increase in pH.^31^ on the other hand, the observation made in stage-IV was contrary to that observed in stages (I), (II) and (III). The pH profile observed herein revealed that anammox reaction in the UPPAR had attained stability (Fig. 2F). To this end, the anammox process conducted in the present UPPAR achieved stability after the startup phase.

### 3.4 Estimation of stoichiometric ratios at start-up and steady-state

The anammox process employed in this study followed the stoichiometric equation given in eqn (6). Consequently, the ratio of NH_4_^+^-N and NO_2_^−^-N removal, as well as NO_3_^−^-N production estimated during the startup phase agreed with established theoretical values associated with anammox reaction (Fig. 2E and Table 1). As illustrated and presented in Fig. 2E and Table 1, respectively, it could be seen that the ratio of ΔNH_4_^+^ : ΔNO_2_^−^ : ΔNO_3_^−^ varied at the proposed operational stages (I, II, III, and IV). In stage (I), the conversion efficiency of NH_4_^+^-N and NO_2_^−^-N was relatively low, hence ΔNH_4_^+^ : ΔNO_2_^−^ : ΔNO_3_^−^ ratio was not apparent (Fig. 2E). The ΔNH_4_^+^ : ΔNO_2_^−^ : ΔNO_3_^−^ ratio estimated in stage (I) revealed that nitrogen removal within the UPPAR was not entirely carried out by anammox bacteria alone but also, other traditional nitrifying and denitrifying bacteria including ammonium oxidizing bacteria (AOB) and NOB as well as heterotrophic bacteria. On the other hand, as the inoculum within the UPPAR gradually adapted to the environment in stage (II), NH_4_^+^-N and NO_2_^−^-N conversion or removal rate changed synchronously, leading to some changes in ΔNH_4_^+^ : ΔNO_2_^−^ : ΔNO_3_^−^ ratio (Fig. 2E). It was observable in stage (II) that, ΔNH_4_^+^ : ΔNO_2_^−^ : ΔNO_3_^−^ ratio was 1 : 0.94 : 0.16 indicating synchronous conversion of NH_4_^+^-N and NO_2_^−^-N ratio was almost 1 : 1. It further suggested that the anammox reaction could not reach its optimum efficiency (Fig. 2E) in the reactor during stage II. However, in stage (III), anammox bacteria activity gradually increased along with further increment in the ratio of NH_4_^+^-N and NO_2_^−^-N as well as ΔNH_4_^+^ : ΔNO_2_^−^ : ΔNO_3_^−^ ratio. On the 63^rd^ day (stage-III), ΔNH_4_^+^ : ΔNO_2_^−^ : ΔNO_3_^−^ ratio estimated was 1 : 1.34 : 0.22 (Fig. 2E), which was much closer to the theoretical value (1 : 1.32 : 026). The ratio estimated in stage (III) confirmed that an optimum or efficient anammox process was fully achieved in the UPPAR by the 63^rd^ day. Consequently, ΔNH_4_^+^ : ΔNO_2_^−^ : ΔNO_3_^−^ ratio at the steady-state (stage IV) was 1 : 1.29 : 0.25, indicating that the UPPAR maintained a steadily complete anammox process throughout the long-term operation (Fig. 2E). Although evaluation of ΔNH_4_^+^ : ΔNO_2_^−^ : ΔNO_3_^−^ ratio in anammox process is not guaranteed to be consistent with theoretical values in most cases, the evaluation exercise, however, gives more insight into anammox bacteria performance which could be used as a guide whilst conducting anammox process. The correlation of ΔNH_4_^+^ : ΔNO_2_^−^ : ΔNO_3_^−^ ratio estimated in this study to theoretical ΔNH_4_^+^ : ΔNO_2_^−^ : ΔNO_3_^−^ ratio was much similar.

Conversely, that estimated and reported by other researchers varied widely. In a study conducted by Feng and coworkers, it was reported that, ΔNH_4_^+^ : ΔNO_2_^−^ : ΔNO_3_^−^ ratio estimated during start-up of anammox biofilm reactor at room temperature was 1 : 1.44 : 0.26.^32^ In another study, Zhou, and coworker reported [ΔNH_4_^+^ : ΔNO_2_^−^ : ΔNO_3_^−^] in a ratio of 1 : 1.54 : 0.3 after successful startup.^33^ Notably, these scholars found that NO_2_^−^-N removal was mostly higher compared to the theoretical value. The variation in [ΔNH_4_^+^ : ΔNO_2_^−^ : ΔNO_3_^−^] ratio of both studies, when compared to the theoretical value, could be as a result of the different inoculum and wastewater (substrate) employed. It is also established that oxygen in the influent matrix, enhances the consumption of NO_2_^−^-N. Consequently, Chen *et al.* used a suspended packed bed biofilm reactor to study the initiation process of anammox process and after a successful start, ΔNH_4_^+^ : ΔNO_2_^−^ : ΔNO_3_^−^ ratio estimated was 1 : 1 : 0.14. Chen found that the consumption of NO_2_^−^-N was lower compared to the theoretical value.^34^ In another similar study, Zhu *et al.*, successfully started up the anammox process with wastewater having ΔNH_4_^+^, ΔNO_2_^−^, and ΔNO_3_^−^ in the ratio of 1 : 1.64 : 0.25, respectively.^35^ In their study, the consumption of NO_2_^−^-N and the production of NO_3_^−^-N were lower than the theoretical values, and this was highly attributed to the dissolved oxygen (DO) concentration in the reactor was high, and the inhibition of anammox bacteria activities that subsequently resulted in the yield of nitrifying bacteria. On the contrary, the amount of NO_3_^−^-N produced in this study was much closer to the theoretical value, although NO_2_^−^-N consumption was relatively lower than the theoretical value.

### 3.5 Characteristics and morphology of sludge

#### 3.5.1 Sludge color and granulation characteristics

**Fig. 3:**
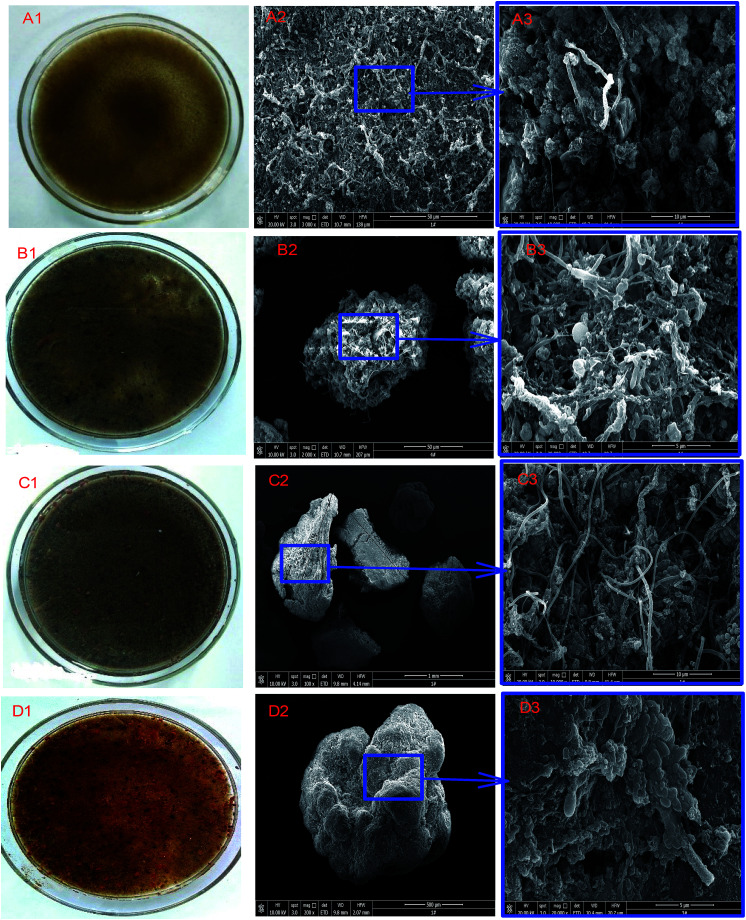
Changes in sludge characteristics and morphology as revealed by SEM: (A1, A2 and A3) inoculum; (B1, B2 and B3) sludge obtained in stage II; (C1, C2 and C3) sludge obtained in stage III; (D1, D2 and D3) sludge obtained in stage IV.

The apparent morphological characteristics (color, granulation, and shape of bacteria) of the inoculum and sludge taken from the UPPAR treating simulated rare-earth wastewater are shown in Fig. 3. As reported in literature, the color of sludge often reveals its viability and performance. It has been established that changes in sludge color from carmine to pale red or black indicates sludge activity reduction.^36^ However, regarding anammox process, Ali and coworkers have indicated that high performing anammox sludge shows a bright red or carmine color. These anammox bacteria contain two key enzymes as a result of a considerable number of Cytochrome *C*. and Heme *C*. cells that are fundamental to the anammox metabolic pathway.^36^ In this study, during the anammox start-up process, sludge shape, texture and color metamorphosed over time. As shown in Fig. 3, the morphology and stain color of the inoculum changed significantly after 124 d of operation. Compared to the yellowish color of the inoculum depicted in Fig. 3AI, the color of the sludge during the various stages of UPPAR operation had changed considerably. At the end of stage-II and stage-III, the respective sludge was darkened (Fig. 3B1 and C1). Countable number small-sized reddish granules together some in the form of flocs were found in the sludge sampled in stage (III). The reddish sludge observed in the sludge indicated the evolution of anammox bacteria (Fig. 3C1). On the hand, after the UPPAR had been operated and attained steady-state (124 days), a significant fraction or volume of the sludge (predominantly granules) seemed more carmine in color with very few dark spots (Fig. 3D1). The predominance of granules in the sludge obtained at stage-IV suggested that the UPPAR promoted the enrichment of anammox bacteria and its granulation simultaneously. The aggregation of sludge flocs into granules with diameter > 200 μm is considered to be particles whereas that with diameter < 200 μm are regarded as flocs.^37^ As illustrated in Fig. 3A2–D2, and Table 3, the sludge obtained in the UPPAR changed significantly over time. The inoculum comprised of about 67.8% of aggregated sludge with size < 0.25 mm, whereas about 0.6% were >2 mm (Table 3). After the UPPAR has been operated for 63 d, it was found that smaller particles aggregated into larger particles with sizes ranging between 1 mm to 2 mm (Table 3). By the end of stage-IV, it was found that a significant fraction of the total volume of flocs sludge had aggregated into granules with sizes predominantly ranging 0.25 mm to >2 mm (Table 3), indicating the UPPAR could promote sludge retention, fluidization and granulation sludge. Notably, at the end of the stable operation, with an average NLR of 1.08 kg m^−3^ d^−1^, larger granular particles were observable (Fig. 3D2 and 4C). Compared to other studies, both granular sludge and the flocculent sludge were observed in the reactor when Cai and coworkers performed studies on start-up in an autotrophic denitrification SBR and EGSB.^38^ In another study, granules were observable after 80 days of operation^39^ indicating the UPPAR employed in this study promoted sludge granulation at a relatively fast rate.

**Table 3:** Distribution of granular sludge with various sizes

Stage	Time (d)	Particle size distribution (%)			
		<0.25 mm	0.25–1 mm	1–2 mm	>2 mm
I	1–6	67.8	24.2	7.4	0.6
II	7–23	nd^a^	nd^a^	nd^a^	nd^a^
III	24–63	45.3	33.2	15.2	6.3
IV	64–124	37.4	24.6	28.3	9.7

a Not determined.

**Fig. 4:**
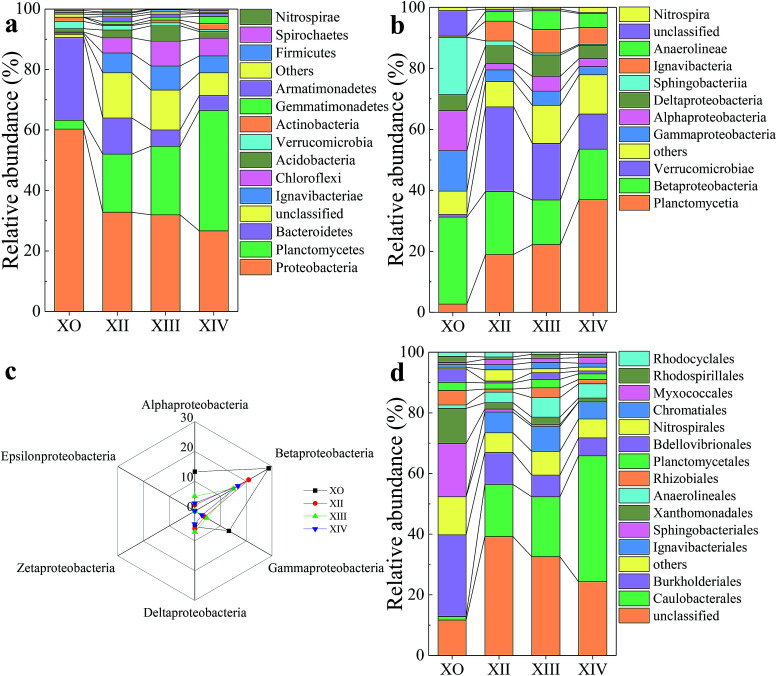
Distribution of microbial community composition (sequence reads ≥1%) at: (a) phylum; (b) class; (c) structure of Proteobacteria at class level; (d) order.

#### 3.5.2 Morphology of sludge within UPPAR

The structure of the sludge granules developed in the UPPAR after 124 days of operation was observed by scanning electron microscopy (SEM). As shown in the SEM micrographs (Fig. 3), coccoid-shaped cells embedded in an extracellular polysaccharide substance (EPS) were observed on the granule surface (Fig. 3A3–D3). The SEM further revealed that the granules contained in the UPPAR mainly had a high degree of compaction with fissures and irregular surfaces (Fig. 3D3). The observation made on the sludge samples with the SEM approach was much similar to that discussed by.^36^ In other studies, Tang *et al.* had reported anammox granule being red-colored and having a cauliflower-like shape^26^ as observed in this study (Fig. 3D2). The surface of the granules obtained from the UPPAR mainly consisted of spherical and elliptical bacteria with few rounded shaped ones (Fig. 3D3). Compared to the inoculum (Fig. 3A3) and the sludge obtained in stages-II (Fig. 3B3), and stage-III (Fig. 3C3), microscopic filamentous bacteria reduced significantly in stage-IV indicating the dominance of anammox bacteria after the entire operation of the UPPAR (Fig. 3D3).

### 3.6 Microbial community evolution and structure

#### 3.6.1 Diversity and richness of the microbial communities

In order to comprehend the nitrogen removal mechanisms conducted in the UPPAR whilst treating simulated rare-earth mining wastewater, microbial community evolution within the reactor was investigated with 16S rRNA high-throughput gene sequencing. With a <0.1% rejection rate as threshold, total quality sequence reads generated by the 16S rRNA gene sequencing for the inoculum (X_0_) and sludge obtained at the end of stage II (X_II_), stage III (X_III_), and stage IV (X_IV_) was 56 500, 59 013, 52 208 and 56 175, respectively (Table 4). Sequence clustering analysis conducted^40^ employed read lengths between 400 and 500 bp for the statistical analysis. With a 97% sequence identity tolerance, the number of OTUs observed among all four samples (X_0_, X_II_, X_III_, and X_IV_) ranged from 869 to 1394 (Table 4). The observable difference between OTU in the inoculums (X_0_) and the sample obtained from the UPPAR at stage (IV) of its operation was statistically significant. All statistical indicators including Shannon inverse, Simpson inverse, Chao1 and ACE indices (Table 4) corroborated with the observations made with OTUs. Thus Shannon and Simpson's inverse revealed that diversity in the X_IV_ was much lesser compared to the inoculum (X_0_).

**Table 4:** Richness, diversity, sequence reads and operational taxonomic units (OTUs) associated with microbial communities of sludge samples at 97% sequence identity

Sludge sample	Sequences	OTU	Shannon	ACE	Chao	Coverage	Simpson
Inoculum	56 500	1124	4.73	1217.33	1176.58	1.00	0.03
End of stage II	59 013	1394	4.84	1665.91	1591.07	0.99	0.03
End of stage III	52 308	1062	4.48	1628.19	1391.12	0.99	0.03
End of stage IV	56 175	898	3.92	1425.58	1250.68	0.99	0.08

As presented in Table 4, it was established that the coverage values of the sludge samples were higher than 99% suggesting the coverage's ability to capture a greater proportion of bacterial diversity as well as indicating a true representation of the microbial composition in the samples (Table 4). ACE and Chao1 indices of X_0_ were lower than that in X_II_, X_III,_ and X_IV_, indicating relative abundances of microbial communities in the reactor were higher than that in the inoculum. The result further suggested that the UPPAR had good hydraulic screening effect.

#### 3.6.2 Taxonomy and relative abundances of the microbial communities

As illustrated in Fig. 4, significant changes were observed in the microbial community structure during and after the startup period. Dominant bacteria affiliated to phylum (Fig. 4a and Table 5) were Planctomycetes (43.52%) followed by Proteobacteria (26.63%), Chloroflexi (5.87%), Ignavibacteriae (5.55%), Bacteroidetes (4.9%), and Acidobacteria (2.16%). At the end of each stage (Fig. 4), some microbial communities gradually decreased or disappeared believed to be either by cell lysis or washout. For example, Bacteroidetes (from 27.47% to 4.9%) and Verrucomicrobia (from 2.42% to 0.62%) were reduced to varying degrees. Compared to latter, Nitrospirae (from 0.52% to 0.01%) population almost disappeared within the UPPAR (Fig. 4). As the UPPAR operation continued, other population of microbial the communities also experienced some level of enrichment. These include Chloroflexi (from 0.36% to 5.87%) and Ignavibacteriae (from 0.4% to 5.55%). Among the abundant phylum observed (Fig. 4a), Proteobacteria and Planctomycetes are the major groups that conduct denitrification activities. However, the dominance of Proteobacteria reduced with time as opposed to Planctomycetes which showed a reverse trend (Fig. 4a). Thus, it could be seen that the proportion of the Planctomycetes in the inoculated sludge was 2.95%, and the proportion of the Proteobacteria was about 60.63% (Table 5), indicating Proteobacteria was the most pronounced microbial community at the time. At steady state, dominant phyla within the microbial communities include Planctomycetes and Proteobacteria with proportions of 43.52% and 26.63%, respectively (Fig. 4a and Table 5). Notably, the Proteobacteria population was reduced by 34% whereas Planctomycetes increased by 40.57%. Correspondingly, the microbial community structure observed at steady-state tied in with the 95% nitrogen removal rate observed in this study. The study reported by Gao and coworkers corroborated with the observation made in this study. Gao *et al.* reported that Planctomycetes increased (from 13.7% to 36.0%) in population whilst conducting a study on the anammox process. On the other hand, Proteobacteria decreased (from 26.6% to 6.0%) significantly.^41^

**Table 5:** Relative abundance of top two phyla affiliated to anammox process within the UPPAR

Sludge sample	Label	Relative abundance of dominant phylum (%)		Total
		Planctomycetes	Proteobacteria	
Inoculum	X_0_	2.95	60.63	63.58
End of stage II	X_II_	19.34	33.07	52.41
End of stage III	X_III_	22.47	31.7	54.17
End of stage IV	X_IV_	43.52	26.63	70.15

**Fig. 5:**
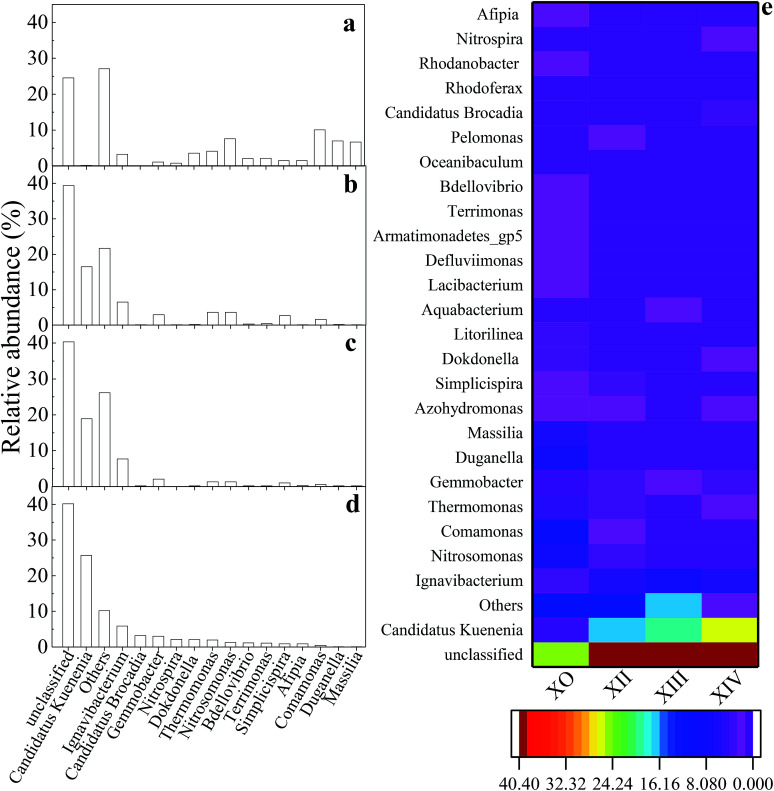
Distribution of microbial community composition at the genus level (sequence reads ≥1%): (a) X_0_; (b) X_II_; (c) X_III_; (d) X_IV_; (e) richness heatmap depicting top 26 predominant genera within inoculum and reactor sludge samples.

As microbial communities ideally contain diverse bacteria and archaea consortium in different proportions, the microbial community analysis was further used to probe for responsible microbes within the UPPAR at class (Fig. 4b and c), order (Fig. 4d) and genus (Fig. 5) level to arrive at a more accurate conclusion. Notably, the dominant bacteria and their respective relative abundances almost agreed with that observed at the phylum level. At the class level, it was found that alpha (α)-Proteobacteria, beta (β)-Proteobacteria, delta (δ)-Proteobacteria, and gamma (γ)-Proteobacteria were the most pronounced sub-groups in league with Proteobacteria (Fig. 4c). The observation tied in with literature where it has been established that most genera responsible for nitrification process (AOB [*Nitrosomonas* and *Nitrosospira*] and NOB [*Nitrobacter* and *Nitrospira*]) are phylogenetically associated with β-Proteobacteria, and γ-Proteobacteria, α-Proteobacteria, γ-Proteobacteria, δ-Proteobacteria.^52^ The predominant class observed among X_0_, X_II_, X_II_, and X_IV_ was (β)-Proteobacteria which accounted for 28.68%, 20.94%, 14.9%, and 16.79%, respectively, of the total population (Fig. 4c). The (β)-Proteobacteria population reduced by significantly by the end of UPPAR operation. This was believed to be a result of NO_2_^−^-N inhibited or wash-out phenomenon and hence giving way to anammox bacteria evolution.

Fig. 5 shows the evolution and composition of the microbial community at the genus level. Dominant bacteria with their respective relative abundances >1% in at least one sample was selected and reported in this study. As illustrated in Fig. 5d, some genera including *Duganella*, *Massilia*, and *Stenotrophomonas*, were eliminated by the end of the UPPAR operation. This observation could be as a result of inhibition caused by the high nitrogen loading introduced in the UPPAR. On the other hand, some new genera such as *Candidatus Kuenenia* and *Candidatus Brocadia* also emerged (Fig. 5d). Studies have shown that anammox bacteria (about 5 types) are microscopically classified as Planctomycetes. The most common anammox genera for anammox are reported as *Candidatus Brocadia* and *Candidatus Kuenenia*.^42^ It can be seen from Fig. 5a that, *Candidatus Kuenenia* and *Candidatus Brocadia* were almost undetected in X_0_. However, the proportion of these two genera (*Candidatus Kuenenia* and *Candidatus Brocadia*) increased as the reactor operation progressed. During the steady-state of the UPPAR operation, *Candidatus Kuenenia* became the dominant bacteria with a relative abundance of 25.64%. In addition, small fractions of *Candidatus Brocadia* was also observable but accounted for only 3.15% (Fig. 5d). Notably, *Candidatus Kuenenia* and *Candidatus Brocadia* accounted for 87.9% of the total genera affiliated to phylum Planctomycetes. Compared to the inoculum (Fig. 5a), it was evident that the relative abundance of *Candidatus Kuenenia* and their growth dynamics was well supported and promoted by the novel UPPAR (Fig. 5d).

The dominance of anammox bacteria observed in this study correlated with the high nitrogen removal from the simulated nitrogen-rich wastewater. According to relevant studies, while *Candidatus Kuenenia* could be more tolerant to nitrite with a concentration of about 180 mg L^−1^, *Candidatus Brocadia* could irreversibly be inhibited by nitrite with concentration over 70 mg L^−1^.^43^ In this study, NO_2_^−^-N concentration in the influent during the steady-state period exceeded 200 mg L^−1^. As a result, *Candidatus Kuenenia* was well enriched by the substrate believing *Candidatus Brocadia* were inhibited by the nitrite concentration employed in this study. Besides the anammox bacteria revealed by the high-throughput gene sequencing, other genera affiliated to phylum Proteobacteria also emerged at the later period of the steady-state (Fig. 5 and Table 5). Studies have demonstrated that some bacteria affiliated to Proteobacteria could promote anammox bacteria growth. Thus filamentous bacteria could build a network-like structure to help the enrichment of anammox bacteria.^44^ Therefore, the observable rapid enrichment of anammox genera within a short period could also be attributed to the support from filamentous bacteria to the Proteobacteria. Also, AOB (*Nitrosomonas*) and NOB (*Nitrospira*) were also found within the UPPAR, but these genera accounted <2.5% of the total population at steady-state (Fig. 5(d)). Since no de-aerator was used in this experiment, it was much likely that the little DO contain in the influent might fostered the growth of the AOB and NOB that was seen in the UPPAR sludge samples (Fig. 5(b)–(d)). Also, *Ignavibacterium* and *Thermomonas* were also observable in the reactor (Fig. 5(b)–(d)). However, the relationship between the dominant bacteria was compared with the GenBank database *via* the BLAST algorithm. It was established that nucleotide sequences obtained in this study were about 83 to 99% similar to published sequences found in the GenBank database (Table 6).

**Table 6:** Relative abundances of genus and closest relatives as revealed by *BLAST* algorithm in GenBank^a^

Accession numbers assigned to gene sequences (in this study)	Relative abundance of the genus in sludge sample (%)				Closest relatives and source in NCBI GenBank [similarity (%)]
	X_0_	X_II_	X_III_	X_IV_	
*Afipia* (MH920340)	1.5	0.1	0.3	0.9	*Afipia birgiae* [99]
*Bdellovibrio* (MH920341)	2.1	0.3	0.2	1.2	*Bdellovibrio exovorus* [98]
*Comamonas* (MH920342)	10.1	1.6	0.6	0.5	*Ottowia shaoguanensis* [98]
*Dokdonella* (MH920343)	3.6	0.2	0.2	2.1	*Dokdonella kunshanensis* [96]
*Duganella* (MH920344)	7.0	0.2	0.2	0.1	*Pseudoduganella danionis* [98]
*Ignavibacterium* (MH920345)	3.3	6.5	7.7	5.9	*Ignavibacterium album* [94]
*Massilia* (MH920346)	6.7	0.1	0.2	0.1	*Massilia phosphatilytica* [98]
*Nitrosomonas* (MH920347)	7.6	3.6	1.3	1.3	*Nitrosomonas eutropha* [97]
*Nitrospira* (MH920348)	0.8	0.1	0.03	2.2	*Nitrospira moscoviensis* [94]
*Rhodanobacter* (MH920349)	1.7	0.2	0.3	1	*Rhodanobacter xiangquanii* [98]
*Simplicispira* (MH920350)	1.5	2.7	1.0	0.9	*Simplicispira psychrophila* [99]
*Terrimonas* (MH920351)	2.2	0.4	0.2	1.1	*Terrimonas lutea* [97]
*Thermomonas* (MH920352)	4.1	0.7	1.3	2.0	*Thermomonas fusca* [96]
*Candidatus Brocadia* (MH920353)	0.1	0.1	0.2	3.2	*Acidicapsa borealis* [83]
*Candidatus Kuenenia* (MH920354)	0.2	16.5	18.9	25.7	*Terriglobus albidus* [84]

a The closest related sequence was established using the NCBI's BLAST platform.

## 4. Conclusions

Immobilization of anammox biomass was proposed using a novel UPPAR and its related contribution to the rapid anammox process start-up and autotrophic nitrogen removal process was investigated. Within 63 days of the UPPAR treating nitrogen-rich rare-earth wastewater, a rapid startup was successfully recorded. Furthermore, over 90% of nitrogen removal was achieved when NLR of 1.08 kg m^−3^ d^−1^ was employed in the UPPAR at the steady-state. After the UPPAR had been operated for 124 d, sludge color changed to carmine, granules size was >2.0 mm and coccoid-shaped cells dominated the surfaces of the granules. High-throughput sequencing revealed that Proteobacteria and Planctomycetes were the most pronounced phylum observed at steady-state, with *Candidatus Kuenenia* (25.46%) and *Candidatus Brocadia* (3.15%) dominating the observed genera within the Planctomycetes. The novel UPPAR was efficient in promoting sludge granulation, anammox bacteria enrichment, and high-autotrophic nitrogen removal.

## Conflicts of interest

The authors of this manuscript have no conflict(s) to declare.

## Supplementary Material
